# The Use of Response Surface Methodology to Optimize the Ultrasound-Assisted Extraction of Five Anthraquinones from *Rheum palmatum* L.

**DOI:** 10.3390/molecules16075928

**Published:** 2011-07-15

**Authors:** Li-Chun Zhao, Jian Liang, Wei Li, Kun-Mu Cheng, Xianghua Xia, Xin Deng, Geng-Liang Yang

**Affiliations:** 1 College of Pharmacy, Hebei University, Baoding 071002, China; 2 The Affiliated Ruikang Hospital of Guangxi Traditional Chinese Medical College, Nanning 530011, China; Email: hyzlc@126.com (L.-C.Z.); 3 College of Chinese Medicinal Materials, Jilin Agricultural University, Changchun 130118, China; 4 Department of Chemistry and Chemical Engineering, Ankang University, Ankang 725000, China

**Keywords:** ultrasound-assisted extraction, anthraquinones, *Rheum palmatum* L, Response surface methodology

## Abstract

In this paper, ultrasound-assisted extraction (UAE) was applied to the extraction of anthraquinones (aloe-emodin, rhein, emodin, chrysophanol and physcion) from *Rheum palmatum* L. The five anthraquinones were quantified and analyzed by high performance liquid chromatography coupled with UV detection (HPLC-UV). The extraction solvent, extraction temperature and extraction time parameters, the three main factors for UAE, were optimized with response surface methodology (RSM) to obtain the highest extraction efficiency. The optimal conditions were the use of 84% methanol as solvent, an extraction time of 33 min and an extraction temperature of 67 °C. Under these optimal conditions, the experimental values agreed closely with the predicted values. The analysis of variance indicated a high goodness of model fit and the success of RSM method for optimizing anthraquinones extraction in *Rheum palmatum* L.

## 1. Introduction

**Figure 1:**
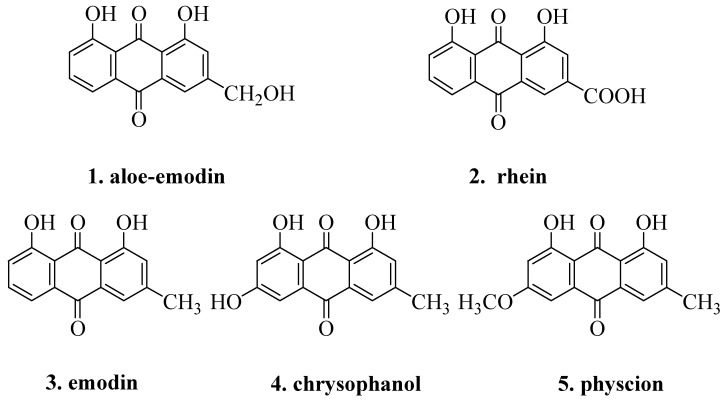
Five anthraquinones in *Rheum palmatum* L.

Rhubarb (Da Huang), one of the most famous and ancient herb medicines, has been used for thousands of years in China. The official rhubarb is described in the Chinese Pharmacopoeia as the dried rhizome and root of *Rheum palmatum *L., *Rheum tanguticum* Maxim. ex Balf., and *Rheum officinale* Bail., [1]. The dried rhizome and root of *Rheum palmatum* L. (Zhangyedahuang in Chinese), the most commonly used species, has many effects such as purgation, purging heat, promoting blood circulation and removing blood stasis [2,3,4]. The main bioactive constituents of *R. palmatum* L. are anthraquinones, including aloe-emodin, rhein, emodin, chrysophanol and physcion (Figure 1) [5,6,7].

The methods commonly used for the extraction of anthraquinone compounds in rhubarb are maceration extraction (ME), heat reflux extraction (HRE), soxhlet extraction (SE) [8,9,10] and microwave-assisted extraction (MAE) [11]. Prior to this study, an ultrasonic humidifier was employed in a UAE method to extract three anthraquinones (aloe-emodin, rhein, emodin) [12]. Perhaps this ultrasonic humidifier was not entirely suitable for the extraction of anthraquinones because of the absence of chrysophanol and physcion. Of course, this incomplete extraction may also be attributed to sample collection. In contrast, UAE methods with conventional ultrasonic cleaners are reported to give better extractions of natural products [13,14,15,16,17]. Many factors affect the extraction efficiency of UAE. Some of these are ultrasonic power, extraction time, extraction temperature, and solvent to solid ratio. The conventional optimization methods usually investigate one variable at-a-time, which is not only time consuming, but also fails to consider the possible interactions between different variables. Response surface methodology (RSM), an effective statistical technique, can optimize complex extraction procedures by investigating the variables and the interactions of the variables simultaneously [18,19]. 

The objective of the present work was to apply response surface methodology to optimize UAE technology for the extraction of five anthraquinones from rhubarb. Several important factors, such as extraction solvent, extraction time and extraction temperature, were systemically analyzed using a Box-Behnken design combined with response surface methodology. 

## 2. Results and Discussion

### 2.1. Model Fitting

Generally, overfitting occurs when a model is excessively complex, such as having too many parameters relative to the number of observations [20,21]. In the present investigation, we selected three main parameters for optimization of the extraction of the five anthraquinones in order to avoid overfitting. Furthermore, preliminary trials were carried out in order to establish a more realistic extraction mode. Finally, the range of methanol concentrations (30-100%), extraction time (10-50 min) and extraction temperature (30-90 °C) was fixed. Table 1 presents the experimental design and corresponding response data for the extraction of the anthraquinones. 

**Table 1:** Box-Behnken experimental design with the independent variables.

Run	Variables levels			Response (Y, mg/g)
	*X*_1_, methanol (%)	*X*_2_, time (min)	*X*_3_, temperature (°C)	
1	30.00	10.00	60.00	13.78
2	100.00	10.00	60.00	16.33
3	30.00	50.00	60.00	16.01
4	100.00	50.00	60.00	16.45
5	30.00	30.00	30.00	14.32
6	100.00	30.00	30.00	15.75
7	30.00	30.00	90.00	15.13
8	100.00	30.00	90.00	16.89
9	65.00	10.00	30.00	13.79
10	65.00	50.00	30.00	15.51
11	65.00	10.00	90.00	15.22
12	65.00	50.00	90.00	16.25
13	65.00	30.00	60.00	17.28
14	65.00	30.00	60.00	17.21
15	65.00	30.00	60.00	17.42

The analysis of variance for the extraction yields of the anthraquinones from the Box-Behnken design is shown in Table 2. The determination coefficient (*R^2^*) of the model is 0.9975, with no significant lack of fit at *P *> 0.05. That means that we were able to explain of 99.75% results using the calculated model. The results indicated that the model used to fit response variable was significant (*P *< 0.0001) and adequate to represent the relationship between the responses and the independent variables [22,23]. The *F*-value, 316.1, implied that the model was highly significant. The adjusted determination coefficient (*R*^2^_adj_), 0.9944, indicated only 0.56% of the total variations were not explained by model. Meanwhile, the coefficient of variation (C.V. = 0.57) of less than 5% indicated that the model was reproducible. 

**Table 2:** Analysis of variance for the fitted quadratic polynomial model of extraction of anthraquinones.

Source	Sum of squares	Degree of freedom	Mean square	*F*-value	Prob > F	
Model	23.97	9	2.66	316.1	< 0.0001	significant
Residual	0.059	7	> 0.01			
Lack of fit	0.031	3	0.01	1.48	0.3466	not significant
Pure error	0.028	4	> 0.01			

Table 3 shows that anthraquinone extraction yield was affected significantly by all three variables (*P *< 0.001). It was evident that all the quadratic parameters (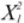, 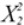, 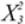) and one interaction parameters (*X*_1_*X*_2_) were significant at the level of *P *< 0.0001, whereas the other interaction parameters (*X*_1_*X*_3_,* X*_2_*X*_3_) were insignificant (*P *> 0.1). The predicted second-order polynomial model was:
*Y *= 17.31 + 0.77*X*_1_ + 0.64*X*_2_ + 0.52*X*_3_ − 0.53*X*_1_*X*_2_ + 0.083*X*_1_*X*_3_ − 0.17*X*_2_*X*_3_− 0.67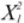 − 1.0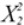 − 1.12
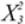
(1)
where *Y* is the yield of five anthraquinones (mg/g), and *X*_1_, *X*_2_ and *X*_3 _are the coded variables for methanol concentration, extraction time and extraction temperature, respectively.

**Table 3:** Estimated regression model of relationship between response variables (yield of four anthraquinones) and independent variables (*X*_1_, *X*_2_, *X*_3_).

Variables	Degree of freedom	Sum of squares	Mean square	*F*-values	*p*-value
*X*_1_	1	4.77	4.77	566.70	< 0.0001
*X*_2_	1	3.25	3.25	385.94	< 0.0001
*X*_3_	1	2.12	2.12	251.87	< 0.0001
*X*_1_ *X*_2_	1	1.11	1.11	132.12	< 0.0001
*X*_1_ *X*_3_	1	0.027	0.027	3.23	0.1153
*X*_2_ *X*_3_	1	0.12	0.12	14.13	0.0071
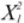	1	1.87	1.87	222.19	< 0.0001
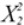	1	4.18	4.18	496.56	< 0.0001
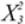	1	5.25	5.25	623.33	< 0.0001

### 2.2. Response Surface Optimization of UAE Condition

**Figure 2:**
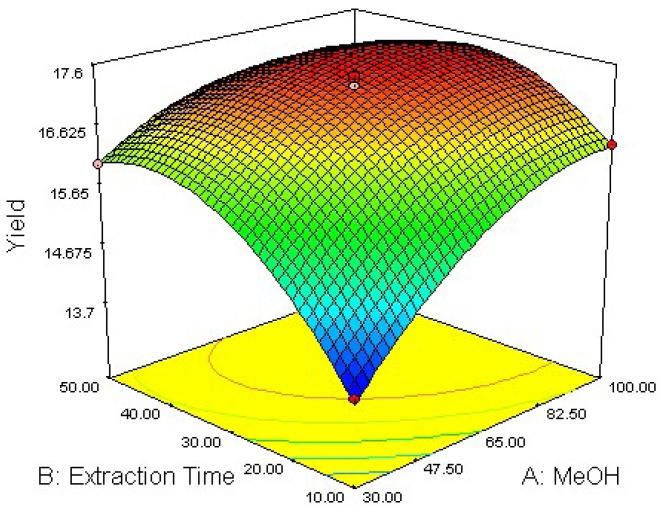
Response surface plot of methanol concentration and extraction time.

**Figure 3:**
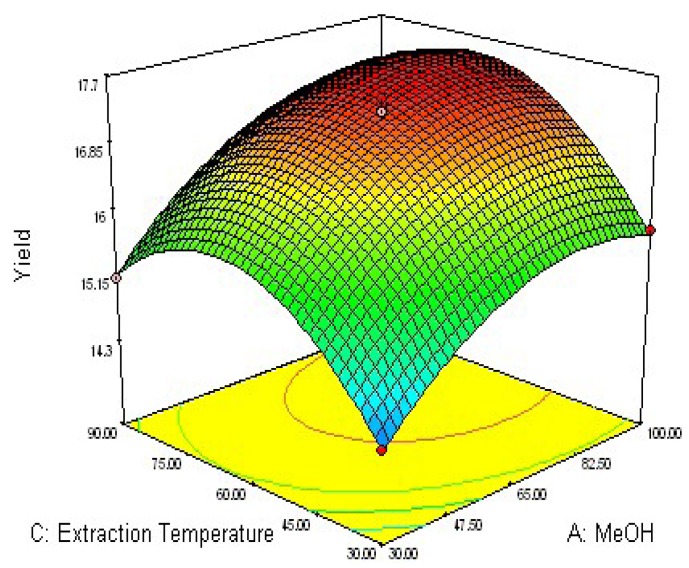
Response surface plot of methanol concentration and extraction temperature.

**Figure 4:**
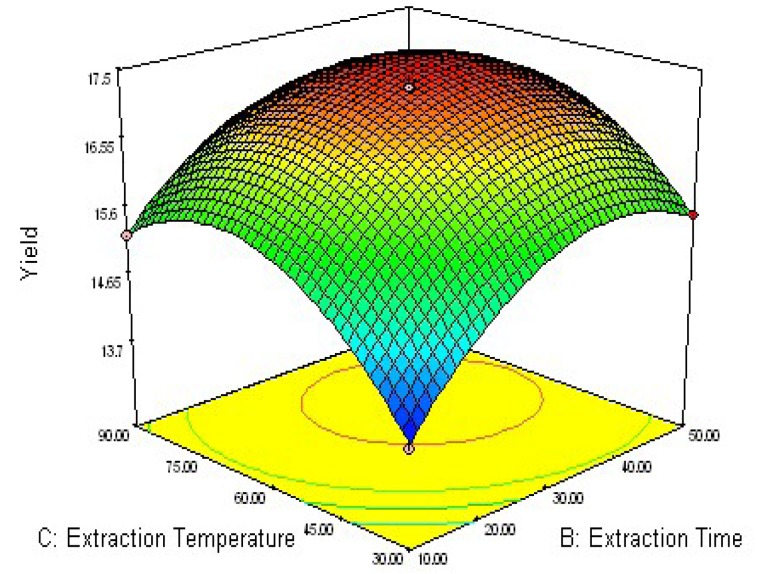
Response surface plot of extraction temperature and extraction time.

To determine optimal levels of the variables for the anthraquinones extraction, the three-dimensional surface plots were constructed according to Equation (1). The effect of the independent variables and their mutual interaction on the yield of anthraquinones in *R. palmatum* L can be seen in Figure 2, Figure 3, Figure 4.

Figure 2 shows the effect of methanol concentration (*X*_1_) and extraction time (*X*_2_) on the yield of anthraquinones in *R. palmatum* L at a fixed extraction temperature. At a definite extraction time, the extraction yield increased slightly with methanol concentration from 30 to 85% and nearly reached a peak at the highest extraction time. However, upon increasing the methanol concentration beyond 85%, there was a gradual decline in the response and extraction times over 33 min did not show any obvious effect on extraction yield. The results may be explained that increasing extraction time may accelerate chemical decomposition of anthraquinones during the extraction process, which leads to the lower extraction yield [24]. 

The effect of methanol concentration (*X*_1_) and extraction temperature (*X*_3_) on the yield of anthraquinones in *R. palmatum* L is shown in Figure 3. Upon increasing the methanol concentration from 30 to 90% with an increase of extraction temperature from 30 to near 85 °C, the extraction yield of anthraquinones increased with methanol concentration. The results are in accord with the data in Table 3, which showed that the interactive effect of methanol concentration with extraction temperature on the yield of anthraquinones was not very weak (*p *= 0.1153).

Figure 4 shows the effect of the interaction of extraction time (*X*_2_) and extraction temperature (*X*_3_) on the extraction yields. The results indicate that the highest extraction yield could be achieved when using 67 °C as extraction temperature and 33 min as extraction time. However, the extraction yield gradually decreased with extraction temperatures over 67 °C. It could be explained that, as extraction temperature increased, more impurities were extracted, resulting in a lower overall yield of anthraquinones [25]. 

### 2.3. Optimization of Extraction Parameters and Validation of the Model

In the present investigation, the software predicted that the optimum methanol concentration, extraction time and extraction temperature were 83.6%, 33.2 min and 67.1 °C, respectively. The software predicted the optimized extraction yield of total anthraquinones to be 17.62 mg/g. Table 4 shows three parallel experiments which were carried out under the optimal conditions, in which the average of extraction yields of total anthraquinones was 17.55 mg/g. Comparing with the value predicted by Design-Expert 8 (trial version), the results show that the predicted value was very close to the actual result, indicating that the optimization parameters proposed in the present are reliable.

**Table 4:** Optimum conditions and the predicted and experimental value of response at the optimum conditions.

	Methanol(%)	Extraction time (min)	Temperature (°C)	Yield of four anthraquinones
**Optimum conditions**	**83.6**	**33.2**	**67.1**	**17.62 (predicted)**
**Modified conditions**	**84.0**	**33.0**	**67.00**	**17.55 (actual)**

## 3. Experimental

### 3.1. Plant Material

The dried rhizome and root of *R. palmatum* L were bought from a drug store in Qianghai Province. The cut pieces were ground to obtain a relatively homogenous powder (0.2-0.5 mm). The powder was dried at 60 °C to a constant weight and was well blended before use. 

### 3.2. Chemicals and Reagents

Methanol was of HPLC grade from Fisher Chemicals (USA). Other chemicals, such as ethanol, *etc*. were all of analytical grade from Beijing Chemical Factory. Water was purified using a Milli-Q water purification system (Millipore, USA). Standards of aloe-emodin, rhein, emodin, chrysophanol and physcion were obtained from the National Institute for the Control of Pharmaceutical and Biological Products of China.

### 3.3. Ultrasound-Assisted Extraction

An ultrasonic cleaning bath (Kunshan Electronics Co, Ltd., China, KQ-250DB) with a frequency of 40 kHz and a maximum peak power of 250 W was used in present study. About 1.0 g of sample were placed in the ultrasonic bath and sonicated at 40 kHz for a certain time at different extraction temperatures. The filtrate was collected and the residue was extracted again (two times) with the same volume of fresh solvent. According to preliminary trials (not reported here), some experimental parameters, *i.e*., methanol as extraction solvent, 0.2-0.5 mm of material size, 1/15 of solid to solvent ratio and 40 kHz of sonication working frequency, were suitable for the process. All the samples were prepared and analyzed in triplicate.

### 3.4. HPLC analysis of Anthraquinones

**Figure 5:**
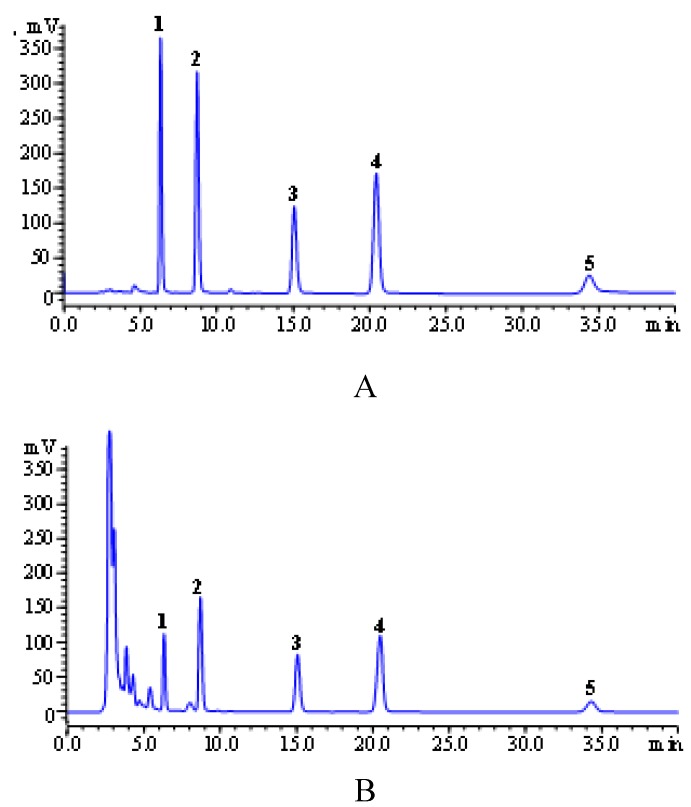
HPLC chromatograms of standard solution of five anthraquinones (**A**); extracts of *Rheum palmatum* L. (**B**); (1) aloe-emodin, (2) rhein, (3) emodin, (4) chrysophanol, (5) physcion.

All anthraquinones in *R. palmatum* L were quantified by high performance liquid chromatography coupled with UV detection (HPLC-UV). The analysis was performed with a HPLC instrument (Agilent 1100, USA) equipped with a quaternary solvent delivery system, a column oven and UV detector. Separation was achieved on Hypersil ODS2 column (4.6 mm × 250 mm, 5 μm) from Dalian Elite Analytical Instruments Co., Ltd. The column temperature was set at 30 °C and detection wavelength was set at 254 nm. The mobile phase was consisted of water containing 0.1% H_3_PO_4_ and 75% methanol (B) with flow rate of 1.0 mL/min. The isocratic elution was employed with 20 μL of injection sample. Figure 5 shows chromatograms from the simultaneous determination of the five anthraquinones in *R. palmatum* L.

### 3.5. Experimental Design

Box-Behnken factorial design (BBD) (Design-Expert software, Trial Version 8, Stat-Ease Inc., Minneapolis, MN, USA) was applied to determine the best combination of extraction variables for the yields of five anthraquinones in *R. palmatum* L. Three extraction variables considered for this investigation were *X*_1_ (methanol concentration), *X*_2_ (extraction time) and *X*_3_ (extraction temperature). The proper range of the three variables was determined on the basis of single-factor anthraquinone production experiments (Table 1). The whole design consisted of 15 experimental points as listed in Table 1, and three replicates (run 13-15) at the center of the design were used for estimating a pure error sum of squares. 

### 3.6. Data Analysis

Data were expressed as standard errors of the means (SEM) of three replicated determinations. The response obtained from each set of experimental design (Table 1) was subjected to multiple non-linear regressions using the Design-Expert software. The quality of the fit of the polynomial model equation was expressed by the coefficient were checked by *F*-test and *p*-value.

## 4. Conclusions

In the present paper, the ultrasound-assisted extraction of anthraquinones from *R. palmatum *L was performed with a three-variable, three-level Box-Behnken design (BBD) based on response surface methodology (RSM). The experimental results showed that all three factors contributed to the extraction of anthraquinones. As such, it may be said that UAE is an effective and indeed feasible method for the simultaneous extraction of the five anthraquinones from *R. palmatum *L.
